# Recurrent cotyledonoid dissecting leiomyoma of the uterus: A case report

**DOI:** 10.1097/MD.0000000000044182

**Published:** 2025-09-05

**Authors:** Fenfen Jiang, Lei Song, Xiaomei Hu, Junjie Qin, Mingshu Zhou

**Affiliations:** a Department of Obstetrics and Gynecology, Electric Power Teaching Hospital, Capital Medical University, Beijing, China; b Department of Obstetrics and Gynecology, Chinese PLA General Hospital, Beijing, China.

**Keywords:** benign tumor, cotyledonoid dissecting leiomyoma, recurrence, uterus

## Abstract

**Rationale:**

Cotyledonoid dissecting leiomyoma (CDL) is an exceptionally rare and morphologically unusual benign uterine leiomyoma. Its malignant-mimicking radiographic and intraoperative features pose a significant diagnostic challenge, often leading to misinterpretation and potentially overtreatment. This case is reported for its rarity and to highlight the critical importance of pathological recognition.

**Patient concerns:**

A 23-year-old female presented with a recurrent pelvic mass detected during a routine follow-up examination 2 years after the initial surgical resection of a uterine mass. The patient was asymptomatic.

**Diagnoses:**

Histopathological examination of both the initial and recurrent masses confirmed the diagnosis of CDL. The specimens exhibited characteristic features including intramural dissecting growth and an exophytic, cotyledon-like nodular appearance.

**Interventions:**

The patient underwent surgical excision for both the primary uterine mass and the recurrent pelvic mass.

**Outcomes:**

The patient recovered well postoperatively with no complications. No evidence of disease was found at the most recent follow-up visit.

**Lessons:**

This case represents the first documented instance of recurrent CDL in China and only the second reported globally. It underscores that recurrence, although exceedingly rare, is a possible outcome for CDL. Pathological confirmation is paramount to achieve an accurate diagnosis, avoid a misdiagnosis of malignancy, and prevent unnecessary radical surgery or adjuvant therapy, thus preserving fertility and quality of life in young patients.

## 1. Introduction

First described by Roth et al in 1996,^[1]^ cotyledonoid dissecting leiomyoma (CDL) is a benign uterine neoplasm characterized by exophytic, placenta-like morphology and dissecting growth through smooth muscle bundles. Only one recurrent case has been reported worldwide,^[2]^ with no prior recurrence documented in China.

## 2. Case presentation

A 23-year-old nulliparous woman presented with an asymptomatic pelvic mass detected during routine examination. Two years earlier, she underwent open resection of a left broad ligament mass and right ovarian cyst enucleation at a tertiary hospital. Initial pathology suggested angiomyoma, with unremarkable 3- and 12-month postoperative ultrasounds. At 27 months postoperatively, ultrasound revealed a 9.9 × 6.4 cm left parametrial mass with heterogeneous echogenicity, well-defined borders, and lobulated contours.

### 2.1. Physical and laboratory findings

Pelvic examination identified a firm, non-tender 10 × 6 cm left parametrial mass. Tumor markers showed isolated elevation of neuron-specific enolase (27 ng/mL; normal <16.3 ng/mL), with normal alpha-fetoprotein, carcinoembryonic antigen, cancer antigen (CA) 125, CA199, and CA724.

### 2.2. Imaging characteristics

**Figure 1.**
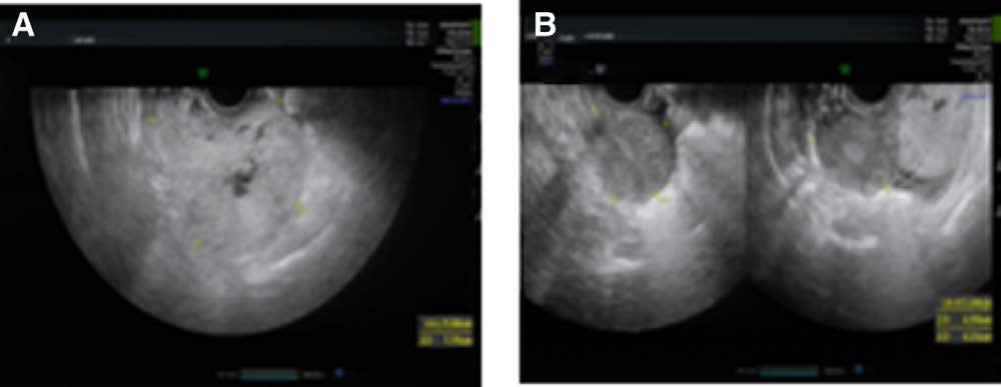
Ultrasonography: (A) a hyperechoic nodule with an indistinct boundary; (B) the nodule was connected to the uterus with an irregular shape.

Ultrasound: 9.1 × 7.7 cm irregular hyperechoic nodule posterior to cervico-uterine junction with ill-defined margins and low vascular resistance (resistive index = 0.50) (Fig. 1A and B).

**Figure 2.**
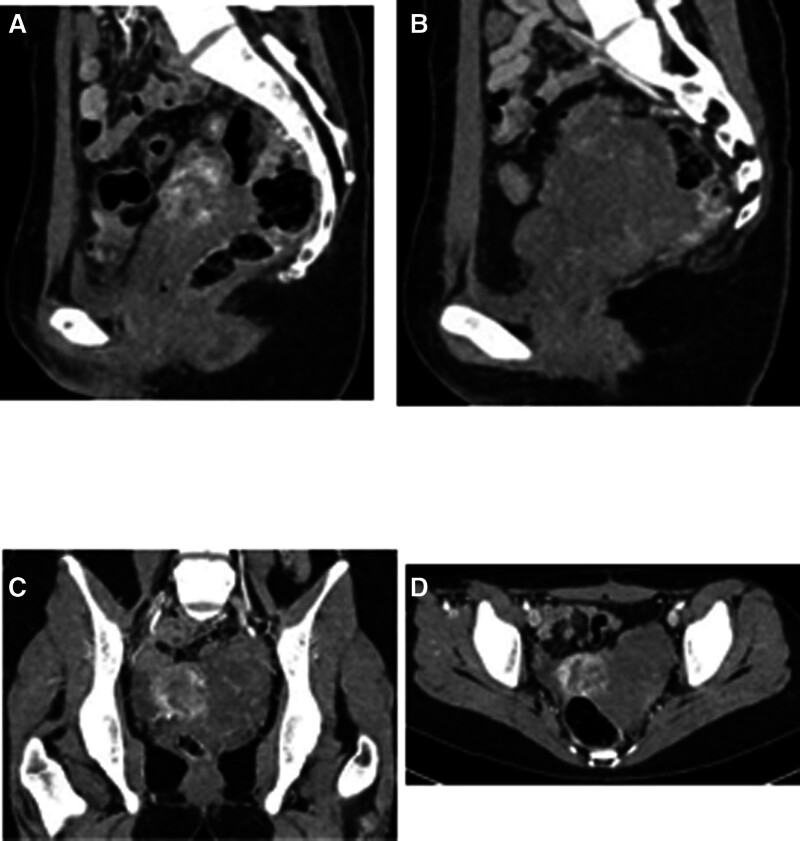
Enhanced CT images: (A) sagittal view showing bilateral ovaries and pelvic occupancy; (B) sagittal view displaying the maximum diameter of the pelvic occupancy in this section; (C) coronal view in the arterial phase; (D) axial view. CT = computed tomography.

Computed tomography: 8.6 × 5.0 cm left adnexal mass with progressive enhancement (46 → 77 HU), traversing vessels, and indistinct cervical borders (Fig. 2A–D).

**Figure 3.**
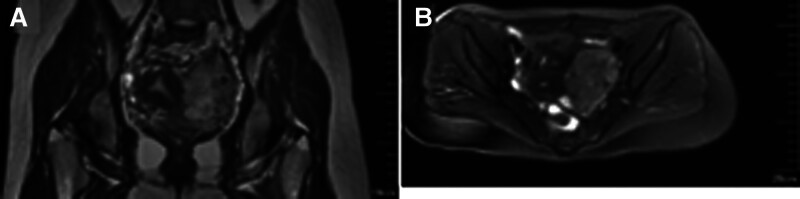
Pelvic MRI: (A) T2-weighted image showing a lobulated appearance of the pelvic occupancy; (B) MRI image of the pelvic occupancy with fat suppression technique applied. MRI = magnetic resonance imaging.

Magnetic resonance imaging: 5.2 × 9.6 × 9.7 cm T2-hyperintense lesion with restricted diffusion, lobulated margins, and mass effect displacing uterus/anterior rectum (Fig. 3A and B).

### 2.3. Surgical findings

**Figure 4.**
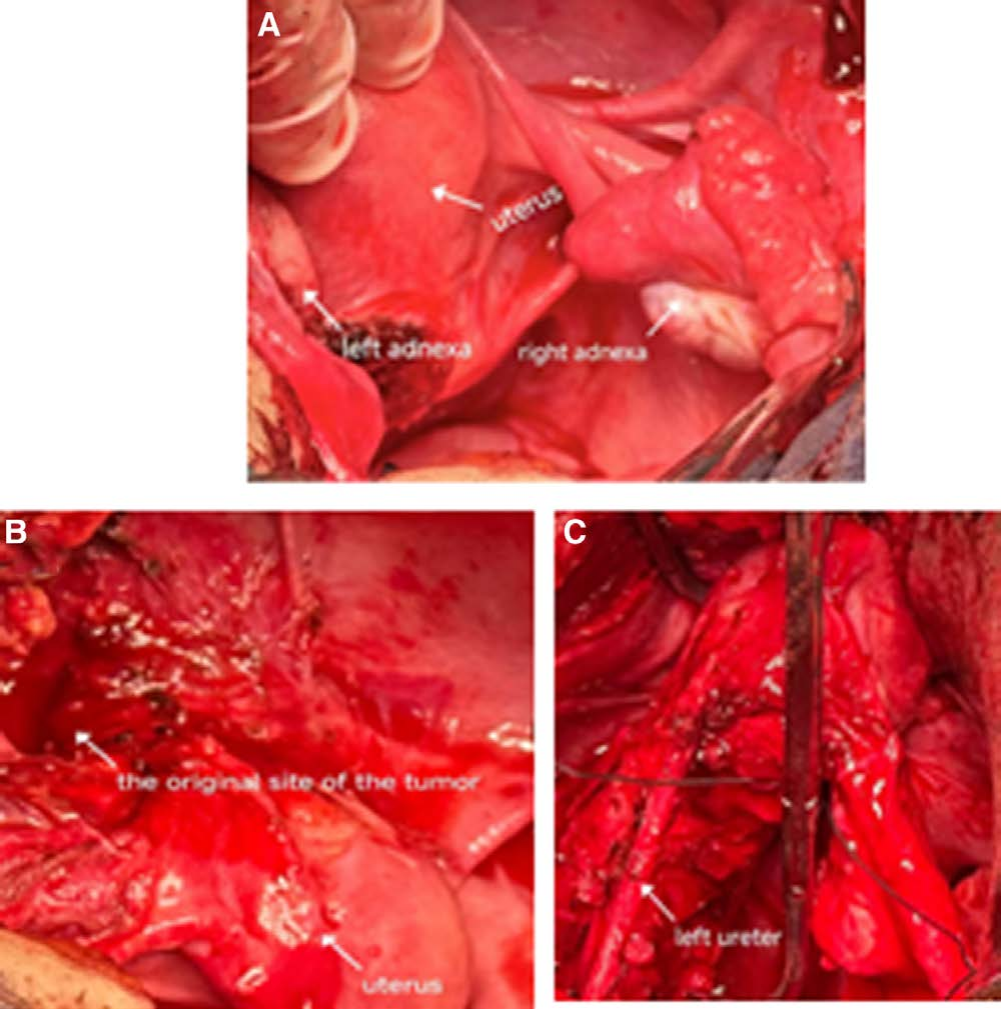
Surgical field: (A) uterus/adnexa; (B) tumor site; (C) dissected left fallopian tube showing mass encasement.

Laparotomy revealed a 10 × 7 cm left broad ligament mass compressing the left adnexa and enveloping the ureter (Fig. 4A–C). The left fallopian tube exhibited blind-ended distortion. Dissection involved significant spurting hemorrhage (estimated blood loss: 1800 mL; transfused: 4U packed red blood cells + 400 mL fresh frozen plasma). The resected mass (10 × 7 cm) showed a beefy, hemorrhagic cut surface with nodular architecture and rubbery consistency. Gross photography was omitted intraoperatively due to unrecognized diagnostic significance. Frozen section suggested benignity.

### 2.4. Pathology

Primary specimen reevaluation: CDL with hemorrhage, congestion, and edema. Immunohistochemistry (IHC): smooth muscle actin (+), Caldesmon (+), and CD34 (vascular+).

**Figure 5.**
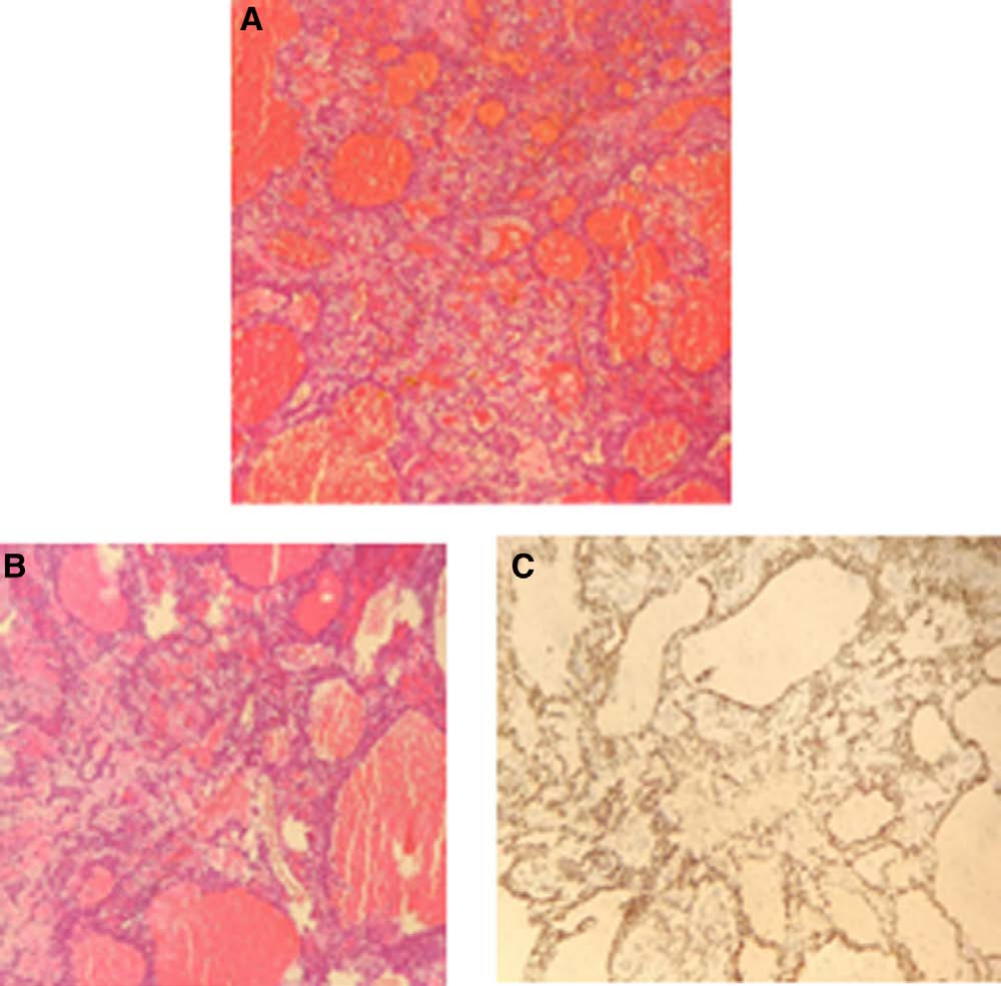
Pathology: (A) dilated blood vessels are visible (HE ×100); (B) edema and dilated blood vessels (HE ×100); (C) positive staining for Desmin (immunohistochemical staining ×100).

Recurrent specimen: hemorrhagic/edematous CDL. IHC: Desmin (+), Ki-67 (10%+), and WT-1 (+) (Fig. 5A–C).

Definitive diagnosis required: absence of endothelial lining in pseudovascular spaces (Fig. 4B); IHC co-expression of Desmin (+) and WT-1 (+); exclusion of coagulative necrosis.

## 3. Discussion

CDL, also known as Sternberg tumor, represents a rare benign variant of leiomyoma, initially reported by Roth et al in 1996.^[1]^ To date, only a scarce number of cases have been documented globally, with a solitary instance of recurrence reported worldwide.^[2]^ Notably, there are no reports of recurrent cases within China. This condition typically affects women in their reproductive years, with an age range of 23–73 years.^[3]^ Clinically, CDL lacks specific symptoms. Ultrasonographic examination reveals one or more lobulated nodules with irregular borders, and in cases where the extracorporeal component of the tumor is substantial, determining its origin can be challenging.^[4]^ Grossly, CDL resembles placental tissue, growing invasively into normal smooth muscle cell bundles.^[5]^ This appearance may mimic uterine malignancies, highlighting the importance of understanding the clinical and pathological features of this tumor for accurate diagnosis and effective treatment.

The microscopic examination of CDL reveals variably sized nodules composed of benign smooth muscle bundles, separated by markedly edematous and vascular-rich fibrous connective tissue. The tumor may exhibit various cellular morphologies characteristic of leiomyomas. This neoplasm typically lacks significant nuclear atypia, mitotic activity, and coagulative necrosis.^[6,7]^

Due to its exceptionally low incidence, CDL is an extraordinarily rare entity. The absence of specific clinical symptoms and pathognomonic imaging features renders preoperative diagnosis challenging, frequently leading to misdiagnosis. Differential diagnosis should primarily include: *Uterine sarcomas*: These malignancies typically demonstrate aggressive behavior, histologically characterized by marked cellular atypia, frequent mitotic figures, coagulative tumor necrosis, and elevated Ki67 proliferation indices.^[8]^ *Intravenous leiomyomatosis*: Intravenous leiomyomatosis is defined by the intravascular proliferation of neoplastic smooth muscle cells, with gross and microscopic evidence of tumor extension within venous structures. In contrast, vascular involvement in CDL manifests as microscopic dissection through vascular walls without true intravascular growth.^[9]^ Initial postoperative pathology in this patient was misinterpreted as vascular leiomyoma due to pseudovascular features caused by tumor-associated edema and hemorrhage. This case provides pathological evidence of CDL’s recurrent potential in young patients. However, there are some study limitations: unavailability of gross specimen images; single-case design restricts generalizability; limited follow-up duration (current 6 months).

## Acknowledgments

We thank all the authors for their contributions in the study.

## Author contributions

**Investigation:** Fenfen Jiang, Lei Song, Xiaomei Hu, Junjie Qin.

**Methodology:** Lei Song.

**Project administration:** Fenfen Jiang.

**Resources:** Xiaomei Hu, Junjie Qin, Mingshu Zhou.

**Supervision:** Mingshu Zhou.

**Validation:** Mingshu Zhou.

**Writing – original draft:** Fenfen Jiang.

**Writing – review & editing:** Fenfen Jiang, Mingshu Zhou.

## References

[1] Roth LM Reed RJ Sternberg WH. Cotyledonoid dissecting leiomyoma of the uterus. The Sternberg tumor. Am J Surg Pathol. 1996;20:1455–61. 8944038 10.1097/00000478-199612000-00004

[2] Roth LM Kirker JA Insull M Whittaker J. Recurrent cotyledonoid dissecting leiomyoma of the uterus. Int J Gynecol Pathol. 2013;32:215–20. 23370645 10.1097/PGP.0b013e318257dff4

[3] Chahkandi M Ataei M Bina AR Mozayani F Fanoodi A. Cotyledonoid dissecting leiomyoma of the uterus: a case report and review of the literature. J Med Case Rep. 2023;17:516. 38102631 10.1186/s13256-023-04271-8 PMC10724900

[4] Zheng L Yu P Zhang W. Cotyledonoid dissecting leiomyoma of the uterus: a rare case and literature review. Int J Gynaecol Obstet. 2023;162:1116–8. 37357396 10.1002/ijgo.14962

[5] Abreu RF Bovolim G Baiocchi G De Brot L. Cotyledonoid dissecting leiomyoma of the uterus: a gross and radiologic malignancy mimicker. Int J Gynecol Cancer. 2023;33:1827–9. 37419515 10.1136/ijgc-2022-004119

[6] Zheng WX Shen DH Guo DH. Gynecological and Obstetric Pathology. Vol. 1. 2nd ed. Science Press; 2021.

[7] Yadav A Raychauduri S Kaur L Wadhwa R Bhardwaj M. An unheard variant of leiomyoma cotyledonoid dissecting leiomyoma: case report. J Midlife Health. 2024;15:197–200. 39610967 10.4103/jmh.jmh_15_24 PMC11601921

[8] Neelayadakshi B Sudha V. Exploring the clinicopathological diversity in sarcomatous transformations in the uterus. Cureus. 2024;16:e62671. 39036252 10.7759/cureus.62671 PMC11258678

[9] Zheng T Huang C Xia Q He W Liu Y Ye H. Intravenous leiomyomatosis in the inferior vena cava and right atrium with pulmonary benign metastasizing leiomyoma secondary to a pelvic arteriovenous fistula: a case report and literature review. Cardiovasc Pathol. 2024;73:107685. 39142442 10.1016/j.carpath.2024.107685

